# A theory on ICER pricing and optimal levels of cost-effectiveness thresholds: a bargaining approach

**DOI:** 10.3389/frhs.2023.1055471

**Published:** 2023-08-24

**Authors:** Mikel Berdud, Jimena Ferraro, Adrian Towse

**Affiliations:** ^1^ Office of Health Economics (OHE), London, United Kingdom; ^2^ Economics Department, University of Buenos Aires, Buenos Aires, Argentina; ^3^ Interdisciplinary Institute of Political Economy, CONICET-University of Buenos Aires, Buenos Aires, Argentina

**Keywords:** cost-effectiveness threshold, dynamic efficiency, social surplus, bargaining, incremental cost-effectiveness ratio (ICER), R&D cost, competition

## Abstract

In many health systems around the world, decisions about the reimbursement of—and patient access to—new medicines are based on health technology assessments (HTA) which, in some countries, include the calculation of an incremental cost-effectiveness ratio (ICER). Decision-makers compare the ICER against a pre-specified value for money criterion, known as the cost-effectiveness threshold (CET), to decide in favour of or against reimbursement. We developed a general model of pharmaceutical markets to analyse the relationship between the CET value and the distribution of the health and economic value of new medicines between consumers (payers) and producers (life science industry developers). We added to the existing literature in three ways: including research and development (R&D) cost for developers as a sunk cost; incorporating bargaining using the Nash bargaining solution to model payer bargaining power from regulation and use of competition; and analysing the impact of a non-uniform distribution of developers R&D costs on the supply of innovation. In some circumstances of bargaining power distribution and R&D cost, we found that using a CET value in HTA decision-making higher than the supply-side CET is socially efficient. Decision-makers should consider adjustable levels of the CET or interpretation of ICERs higher than the CET according to the bargaining power effect. The findings of this research pointed to the need for more research on the impact of bargaining power, how R&D investment responds to rewards, i.e. the elasticity of innovation, and pre- and post-patent expiry modelling.

## 1. Introduction

In many health systems, decisions about reimbursement of—and patient access to—new medicines are based on health technology assessments (HTA) which, in some countries, included the calculation of an incremental cost-effectiveness ratio (ICER). Decision-makers compare the ICER against a pre-specified value for money criterion, known as the cost-effectiveness threshold (CET) to decide in favour of or against reimbursement.

Despite its growing importance, there is no agreement on how the CET value should be set and how CET should be used by decision-makers (1). Two main approaches are discussed in the literature, although HTA decision-making using CETs does not necessarily take either of these approaches and, even when it does, may or may not be evidence-based. These two approaches are:

• *Demand-side* approaches, using a CET reflecting the society's monetary valuation of the maximum willingness to pay (WTP) per incremental unit of health gain, also termed as the consumption value of health. This may vary by social context. Its advocates argue that this approach is in line with those taken elsewhere (2, 3). In the UK, for example, the Department for Transport and other government agencies use WTP-based safety values in cost–benefit analyses (4).
• *Supply-side* approaches, assuming the health budget is fixed in the short run and identifying the CET as the shadow price of the budget constraint, which is the maximum ability to pay per unit of health gain given the cost for the health system to produce a unit of health at the margin—a proxy for a measure of the quantity of health displaced when new technologies are funded. Under this approach, if the objective of the system is to maximise population health, the society would never be willing to pay an amount higher than the CET, as health foregone exceeds health gained (5–7). However, this approach requires a set of assumptions to hold, including a fixed health budget; the existence of an objective measure of the health gain [such as the quality-adjusted life year (QALY)]; a judgement to treat all units of health as being of equal weight (“a QALY is a QALY is a QALY”); and prices of medicines included in the ICER calculation to remain unchanged throughout their life cycle.

CETs used in HTA decision-making regulate the prices for new health technologies by setting the threshold for the maximum acceptable price. Pricing new medicines using this ICER–CET mechanism is part of a family of value-based pricing mechanisms (8, 9). We refer to this mechanism as *ICER pricing* where the developer prices the new health technology at the maximum price the CET allows, which is the price that equates the CET and the ICER.

In this paper, we formalised and extended a model of pharmaceutical markets proposed by Paulden (10) and Pandey et al. (11) to analyse the relationship between the CET value and the distribution of the health and economic value of new medicines (economic surplus) between consumers (payers) and the life science industry (developers). We measured the developer surplus as the economic profit and the consumer surplus as the economic value of the net health gained by using the new medicine. The main contribution of our model to the framework proposed by Paulden (10) and Pandey et al. (11) is to incorporate bargaining power—the ability of the payer and the developers to extract surplus from a transaction—using the Nash bargaining solution (NBS). This enables us to assess the impact of different degrees of bargaining power on the distribution of the economic surplus from new medicines. Paulden (10) assumed that the developers have all the bargaining power; hence, they will price new technology using an ICER equal to the CET.

Paulden (10) analysed some cases where manufacturers do not supply because the price allowed by the threshold does not cover their costs. However, sunk research and development (R&D) costs were not specifically incorporated into the model. We included (sunk) R&D costs in the developers' surplus function as a key determinant of the new health technology supply. Consideration of R&D cost as sunk has dynamic implications, with manufacturers that are willing to supply already-developed medicines in the short term at prices exceeding manufacturing costs even if they offer low (or negative) returns from R&D investment but changing long-term R&D investment decisions impacting future innovation. The implications of our model compare with the policy objective “Maximize consumer surplus, subject to consumer and producer surplus each being non-negative” [see p. 30 of Paulden (10)] but allow a discussion of the dynamic effects of such a policy.

A final addition to our model is to allow a non–linear-shaped supply of new health technologies. The effect of incorporating non-uniform distributions of developers’ reserve ICERs results in non-linear supply curves of health technology innovation, discussed in Appendix A.3. We explore the implications of our additions and discuss the optimum CET level from a societal perspective.

Our model is then able to assess the effects of the CET on the supply of, and the demand for, new medicines. It also assesses how the surplus (value) of new medicines might be distributed between payers and developers, allowing us to analyse the dynamic effects of the CET level on the payers' and developers' surplus.

The paper is organised as follows: Section 2 describes the model, Section 3 explains the functioning of the market under our model based on the different cases presented above, Section 4 discusses the policy implications, and Section 5 provides the concluding remarks.

## 2. The model

### 2.1. Baseline assumptions

We begin with a set of baseline assumptions defining a framework that proxies publicly funded health systems using both cost-effectiveness analysis and CETs to inform resource allocation decisions. These are as follows:

1. There is an efficient, single-payer, publicly funded health system that delivers maximum health benefits for patients within a fixed budget. By efficient, we mean that the budget is allocated such that the cost-effectiveness of funded interventions is superior to any unfunded interventions.
2. There is an accepted measure of benefit *h_i_* for per-patient health gains from manufacturer *i*'s medicine.
3. There is a maximum ability to pay for the health system determined by opportunity cost *k*, which is the marginal cost of production of the least cost-effective intervention provided by the health system and funded within the budget, i.e. the shadow price of the production of health.
4. A *policy threshold* or CET $\bar{\lambda }$ is publicly set by a health system decision-maker (the payer), which is set equal to or lower than the opportunity cost, $\bar{\lambda }\leq k$. New technologies are adopted if and only if the ICER is equal to or lower than $\bar{\lambda }$. If $\bar{\lambda }<k$, the payer gets the surplus created by the difference between $\bar{\lambda }$ and *k*.
5. Manufacturers of new technologies do not face generic/biosimilar competition during the patent term.
6. Each developer has a minimum “reserve price”, or “reserve ICER” ${\underline{\lambda }}_{i}$. This is the minimum price that the innovator is willing to accept to supply a new technology. Developers' reserve ICERs are distributed between zero and *λ^M^*, where *λ^M^* represents the reserve ICER of the costliest technology.
7. *λ^M^* is higher than *k*, which is the health system's opportunity cost. As we do not consider budget changes in the model, we take *k* to be exogenous.
8. The developers and the payer have bargaining power over price setting. The weight parameters *β* and 1−*β* measure the developers’ bargaining power and the payer's bargaining power, respectively (with β∈[0,1]). We make the simplifying assumption that the payer's and developers' bargaining power distribution is homogeneous across all technologies under negotiation.
9. New technologies are costly to develop and produce once developed. The manufacturers’ objective is to maximise returns on investment in health technology R&D. In the short run, *λ^M^* depends on the variable unit costs of manufacturing and distribution, as manufacturers will supply developed products at any price equal to or higher than this, although this may not be sufficient to recover the sunk R&D costs. In the long run, R&D costs also matter.
10. *R_i_* is the R&D cost of the new health technology. All R&D is assumed to occur prior to launch.
11. Each new technology is independent, with a different developer. Each developer produces only one technology.
12. The unit manufacturing and distribution cost of a new health technology is *c_i_*, with *Q_i_* being the quantity of medicine sold.
13. Manufacturers of funded interventions that are displaced by the adoption of a newly funded intervention do not reduce the prices to avoid displacement.
14. The model analyses a single-country market with no impact from global markets or international trade.

Assumptions 1–6 are the baseline assumptions either taken or adapted from Paulden (10). We introduce assumptions 7–10 to develop the model. Assumptions 11–14 are the simplifying assumptions we introduce to frame the analysis and scope of results.

### 2.2. The timing of the model

The sequence of players’ decisions and the associated outcomes is separated into five different stages:

− **Stage 1:** nature fixes the value of *k*, the system's opportunity cost.
− **Stage 2:** the developer believes that the system's opportunity cost (*k*) will drive the CET and chooses to invest in R&D, or not, based on its own expectations about the reserve ICER $\left({\underline{\lambda }}_{i}\right)$ required by the R&D investment.
− **Stage 3:** the payer commits to a CET level $\bar{\lambda }$ to inform reimbursement recommendations, which are guided by its health maximisation objective and knowledge of *k*.
− **Stage 4:** the developer sets the initial product price and therefore its ICER.
− **Stage 5:** the final net price of the new technology and corresponding effective ICER or *λ^E^* are set following a bargaining process at the procurement stage. This final price can be equal to or lower than the reimbursement threshold of Stage 3 $\left(\lambda^{E}\leq \bar{\lambda }\right)$.
− **Stage 6:** the medicine is sold (or not) by the developer to the payer and provided (or not) to the patients.

**Figure 1:**
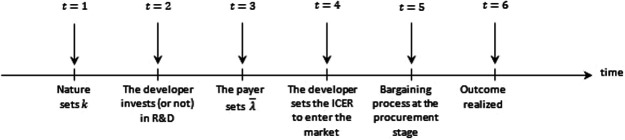
Timing stages of the model.

Figure 1 shows the sequence of stages of the bargaining process in the market for new medicines.

Behaviour at each stage will anticipate behaviour in the next stage. This model timing is the simplest that allows us to analyse the effect of bargaining power distribution, incorporation of the R&D sunk cost, and non-linear supply curves for innovation. It is important to note that if we switched Stages 2 and 3, the developer's decision on R&D investment and the payer's decision on the CET level become endogenous. This alternative setting offers an insightful framework but adds excessive analytical complexity, and we do not pursue this option.

### 2.3. Micro-level behaviour: the Nash bargaining solution (NBS)

In this section, we characterise the reserve ICER of an individual developer and the CET for the payer by defining developer and payer payoff functions, i.e. the profit level and the monetary valuation of society's health gain, respectively. These measure the payer and developer surplus for every price of the medicine. We incorporate bargaining using the Nash bargaining solution (NBS) to calculate the final medicine prices and surplus distribution. Payoff functions also define the set of possible payer–developer agreements.

The following equations represent the profit function of the developer $\Pi_{i}(\lambda^{E})$ and the net population benefit of the payer $\mathrm{NPB}(\lambda^{E})$, both expressed as a function of the agreed ICER of the medicine after the bargaining process $\lambda^{E}\in [\;{\underline{\lambda }}_{i},\bar{\lambda }]$:

(1) $$\Pi (\lambda^{E})=((\beta \bar{\lambda }+(1-\beta ){\underline{\lambda }}_{i})h_{i}-c_{i})Q_{i}-R_{i}$$

(2) $$\mathrm{NPB}(\lambda^{E})=(\left(1-\beta )(\bar{\lambda }-{\underline{\lambda }}_{i})+(k-\bar{\lambda }\right))h_{i}Q_{i}$$

Equations 1 and 2 measure the producer and consumer surplus, respectively, in monetary terms. Equation 1 calculates the producer (developer) profit. The first addend measures the profit level when *R_i_*, i.e. the R&D cost, is excluded. It is calculated by multiplying the price per unit less the variable cost per unit *c_i_* by the number of units sold *Q_i_*. The price per unit is expressed as a function of the reserve ICER ${\underline{\lambda }}_{i}$, the CET $\bar{\lambda }$, the distribution of the bargaining power *β*, and the health gains of the new medicine *h_i_*.

Equation 2 measures the consumer (payer) surplus as the net population benefit (NPB). It is defined as the monetary value of the health benefit provided by new technologies, which is the net monetary value of the health foregone by the displacement of existing technologies. The NPB uses the system's opportunity cost as the monetary value per unit of health gain and, by definition, implies that the payer surplus is strictly positive if and only if the effective (post-bargaining) CET (*λ^E^*) is lower than the opportunity cost of the health system. Note that NPB is conceptually different from net monetary benefit (NMB) which measures the monetary value of the incremental health gains of the new technology using the approval norm $\bar{\lambda }$ instead of the opportunity cost *k*.

The term within brackets captures how much surplus the payer can extract *via* price setting and negotiation. It is a function of the difference between the CET $\bar{\lambda }$ and the maximum ability to pay *k* and the additional surplus extracted by the payer from the difference between the CET $\bar{\lambda }$ and the producer's reserve ICER ${\underline{\lambda }}_{i}$ depending on its bargaining power (1−*β*). This is multiplied by the health gains *h_i_* and the number of units of the new medicine sold *Q_i_*. Detailed formal development of both functions is provided in Appendix A.1.

We assume the total social surplus (TS) results from adding the two individual profit functions:

(3) $$\mathrm{TS}(\lambda^{E})=\Pi_{i}(\lambda^{E})+\mathrm{NB}(\lambda^{E})$$

To show how the payoffs and surplus distribution depend on bargaining power, β∈[0,1], we set out below three cases for the *β* values:

1. When *β* = 1, the developer holds all bargaining power and captures all surplus during the patent period. The agreed ICER is equal to the health system’s maximum ability to pay, $\lambda^{E}=\bar{\lambda }\;$, which corresponds to a price $p_{i}(\bar{\lambda })=\bar{\lambda }h_{i}$. The payer's payoff is $\mathrm{NPB}(\bar{\lambda })=\underline{\mathrm{NPB}}=(k-\bar{\lambda })h_{i}Q_{i}\;$, and the developer's payoff is $\Pi_{i}(\bar{\lambda })=(\bar{\lambda }h_{i}-c)Q_{i}-R_{i}$.
2. When *β* = 0, on the contrary, the payer holds all bargaining power and captures all the surplus. The agreed ICER is equal to the reserve ICER of the developer, $\lambda^{E}={\underline{\lambda }}_{i}$, which corresponds to a price $p_{i}({\underline{\lambda }}_{i})={\underline{\lambda }}_{i}h_{i}=c_{i}$, or the minimum short-term price at which the developer is willing to sell a positive amount. The payoff to the developer is $\Pi_{i}({\underline{\lambda }}_{i})=-R_{i}$. The payer gets $\mathrm{NPB}({\underline{\lambda }}_{i})=\bar{\mathrm{NPB}}=\;(k-{\underline{\lambda }}_{i})h_{i}Q_{i}$.
3. When β∈(0,1), the total surplus is distributed between the payer and the developer. The distribution is determined by relative bargaining power defined by *β*.

Table 1 sets out some results for the *λ^E^* values corresponding to the different bargaining power levels.

**Table 1:** Bargaining power of players.

Developer's bargaining power, *β*	Payer's bargaining power, 1 − *β*	Effective ICER, *λ^E^* (*β*)	Developer's profit: Π*i* (*λ^E^*)	Payer's surplus: *NB* (*λ^E^*)
1	0	$\bar{\lambda }$	$(\bar{\lambda }h_{i}-c_{i})Q_{i}-R_{i}$	$(k-\bar{\lambda })h_{i}Q_{i}$
0.75	0.25	$0.75\bar{\lambda }+0.25{\underline{\lambda }}_{i}$	$((0.75\bar{\lambda }+0.25{\underline{\lambda }}_{i})h_{i}-c_{i})Q_{i}-R_{i}$	$(k-{\underline{\lambda }}_{i}-0.75(\bar{\lambda }-{\underline{\lambda }}_{i}))h_{i}Q_{i}$
0.5	0.5	$0.5\bar{\lambda }+0.5{\underline{\lambda }}_{i}$	$((0.5\bar{\lambda }+0.5{\underline{\lambda }}_{i})h_{i}-c_{i})Q_{i}-R_{i}$	
0.25	0.75	$0.25\bar{\lambda }+0.75{\underline{\lambda }}_{i}$	$((0.25\bar{\lambda }+0.75{\underline{\lambda }}_{i})h_{i}-c_{i})Q_{i}-R_{i}$	$(k-{\underline{\lambda }}_{i}-0.25(\bar{\lambda }-{\underline{\lambda }}_{i}))h_{i}Q_{i}$
0	1	${\underline{\lambda }}_{i}$	$-R_{i}$	$(k-{\underline{\lambda }}_{i})h_{i}Q_{i}$

Examples of agreed incremental cost-effectiveness ratio (ICER) and payoffs.

**Figure 2:**
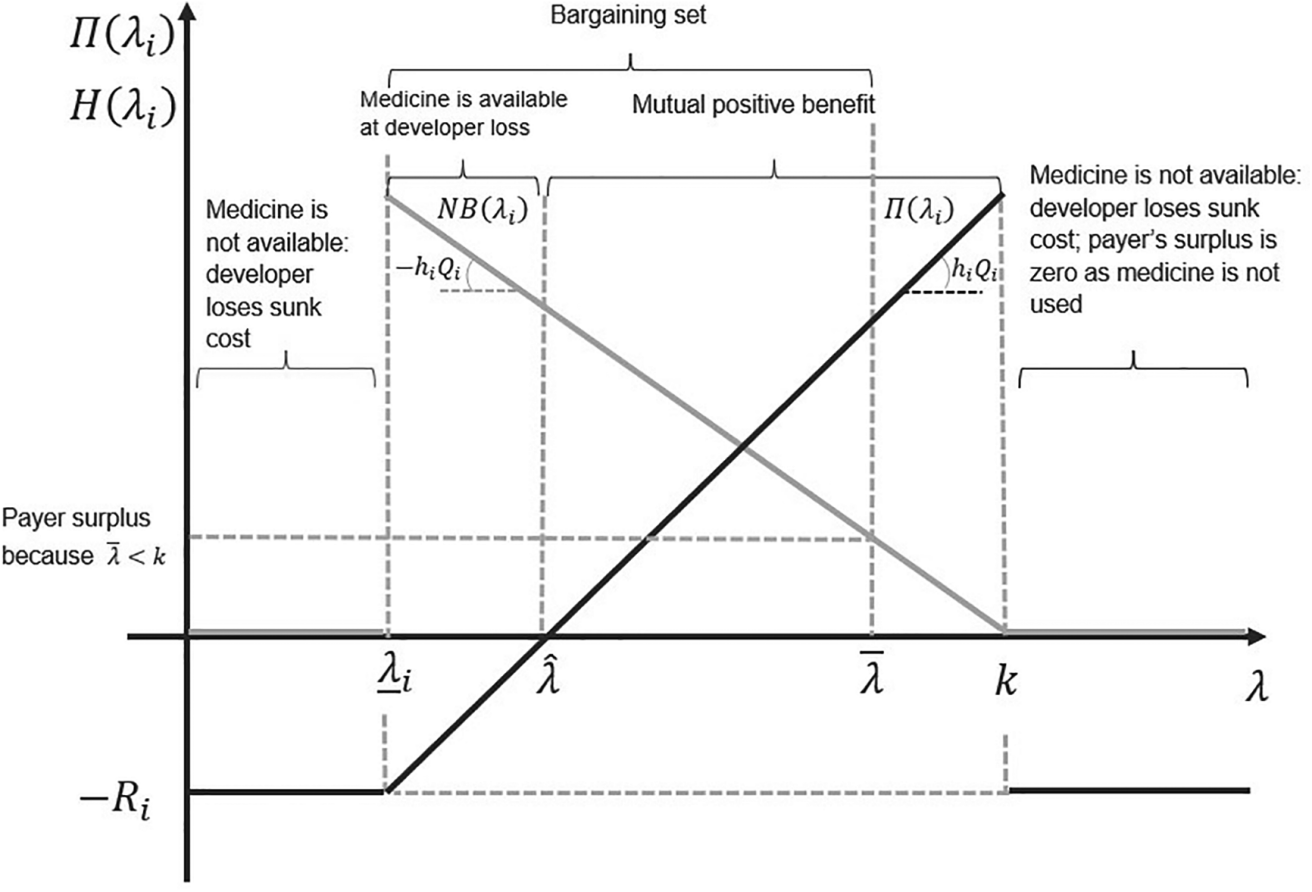
The set of all possible agreements on the incremental cost-effectiveness ratio (ICER). The light grey line represents the payoff to the payer, and the black line represents the payoff to the developer. The slope of the developer's payoff function is the health gain per patient treated times the number of patients treated. The slope of the payer's payoff function is minus the health gain per patient treated times the number of patients treated.

Figure 2 illustrates the bargaining problem. The long-term reserve ICER is the *x*-axis cutting point $\hat{\lambda }$, representing the ICER level where the developer recovers all its R&D investment. If the ICER level is below $\hat{\lambda }$ and above ${\underline{\lambda }}_{i}$, the developer will still sell a developed medicine, albeit at a loss (only a portion of the sunk R&D cost will be recouped). In the short term, ${\underline{\lambda }}_{i}$ is the minimum ICER at which the developer sells a developed medicine.

Other parameters, such as the CET level $\left(\bar{\lambda }\right)$ and the sunk R&D cost (−*R_i_*), are also relevant to our results. We consider these below.

### 2.4. Macro-level implications of the ICER pricing

In the previous section, we characterised the payoff functions of the payer and a single developer, illustrating how bargaining power affects the surplus distribution between them. We now focus on the aggregate supply of innovative medicines, assuming a continuum of developers supplying innovation at their reserve ICERs, distributed between zero and *λ^M^*.

#### 2.4.1. The aggregate demand for innovative medicines: payer perspective

To understand aggregate demand for new medicines, we focus on the macro-level payer utility function—the overall utility that the payer gains by using the healthcare budget to provide and finance health services to patients.

In our ICER pricing model, the overall utility of the payer—while the medicine is on-patent—depends on the following factors: the maximum WTP of the payer, as measured by *k* defined in Stage 1, the CET level fixed by the payer in Stage 3, the ICER at which the developer prices the new technology for reimbursement in Stage 4, and the market outcome resulting from the Stage 5 bargaining process.

We assumed a fixed payer budget, implying that the adoption of a technology necessarily displaces others already in use in the health system. The maximum ability to pay must reflect the health system opportunity cost *k*. Paying more implies that health foregone exceeds health added which decreases the total health produced. Paying less than *k* means that more health is added than health foregone—a net contribution to the payer surplus. In our model, the payer sets the CET with a maximum acceptable level of *k*. A CET below *k* is a policy option for the payer.

Whether the threshold that maximises health production is at *k*, or below it, depends on how much medical innovation developers generate at each threshold. This in turn depends on the developers' reserve ICER distribution. With threshold values closer to *k*, developers with higher reserve ICERs enter the market. More innovation is supplied, but the health gains delivered by new medicines are priced higher. This implies the marginal net health gain per new health technology adopted (the surplus) earned by the payer decreases. The overall result of increasing CET on the payer health gain depends on which of the two effects dominates: the increase in total health gain obtained through more cost-effective technologies entering the market or the decrease in the net health gain per adopted technology due to the higher price paid per unit of health gain.

Given that technologies with ICERs higher than *k* will not be considered, only those whose reserve ICERs are in the interval [0, *k*] will be relevant.

Let the relationship between the net health gain and the CET level be measured by the following linearly decreasing function:

(4) $$h(\bar{\lambda })=a-a\frac{\bar{\lambda }}{k}$$

Parameter *a* measures the maximum health gain achievable for the payer when a medicine's price equals its marginal cost. A CET level equal to the payer's maximum ability to pay $\bar{\lambda }=k$ implies that the net health gain during the patent period is zero. Equation 4 measures the marginal net health gain for the system by adopting the new treatment for every threshold level below or equal to the opportunity cost $\left(\bar{\lambda }\leq k\right)$.

We assume initially that the developers’ reserve ICERs $\underline{\lambda }$ are uniformly distributed along the interval $\underline{\lambda }\in [0,\lambda^{M}]$. By definition, the NPB turns negative if the CET is set above the opportunity cost $\left(\bar{\lambda }>k\right)$ as this allows the adoption of technologies which means more health foregone than added.

We can plot the relationship between the payer's CET and the value of the payer's NPB using an NPB curve with the following function:

(5) $$\mathrm{NPB}=(1-\beta )\int_{0}^{\bar{\lambda }}h(\lambda )d\lambda +\beta \bar{\lambda }h(\bar{\lambda })$$

where *β* is the bargaining power of the developer, $\bar{\lambda }$ is the CET fixed by the payer, and *λ* is a variable that represents any CET value.

By substituting Equation 4 into Equation 5 and solving the integral, we have:

(6) $$\mathrm{NPB}=(1-\beta )\bar{\lambda }\left(a-\frac{a}{2k}\bar{\lambda }\right)+\beta \bar{\lambda }\left(a-\;\frac{a\bar{\lambda }}{k}\right)$$

By applying the first-order conditions to Equation 6 with respect to $\bar{\lambda }$ and rearranging, we obtain:

(7) $$ak=a\bar{\lambda }(1+\beta )$$

Equation 7 shows that the payer's surplus is maximised where the marginal benefit of the last new technology accepted after an increased CET is equal to the marginal cost to the payer of the higher CET. An increased CET means more new technologies are adopted. These new adoptions increase NPB as they provide health benefits to patients (volume effect). On the other hand, an increased CET means the payer pays more per unit of health gain (price effect) as the payer is not able to price discriminate. An increased CET increases the prices paid for all new interventions.

The size of the price effect on the payer is higher for higher values of developer bargaining power *β* as this increases the final price paid for new technologies. At some point, the price effect dominates the volume effect, and the optimal value for the CET is lower. Lower *β* values decrease the final price which increases NPB, allowing the volume effect to dominate the price effect and the optimal value for the CET to be higher. The NPB curve reaches its maximum at a CET such that the volume effect is equal to the price effect. This value for the CET is calculated by solving Equation 7 for the value of $\bar{\lambda }$:

(8) $${\bar{\lambda }}^{*}=\frac{k}{1+\beta }$$

The value of ${\bar{\lambda }}^{*}$ is increasing in *k*. We can note that, as well as the impact of *β* discussed above, an increased *k* means that the payer's maximum ability to pay for a unit of additional health benefit is higher, and therefore, the level of its surplus maximising CET is higher.

#### 2.4.2. The aggregate supply of innovative medicines: industry perspective

The industry-level producer surplus is the sum of all individual developer surpluses (DS*_i_*). The formal expression is:

(9) $$\mathrm{DS}=\beta \left[\int_{0}^{\bar{\lambda }}\left(a-\frac{a}{k}\lambda \right)d\lambda -\bar{\lambda }\left(a-\frac{a}{k}\;\bar{\lambda }\right)\right]-R$$

The first addend of the equation (in brackets) represents the share of the total surplus that the industry obtains given its bargaining power *β*. The second addend represents the total R&D cost incurred by all developers. By rearranging Equation (9), we have:

(10) $$\mathrm{DS}=\frac{\beta a{\bar{\lambda }}^{2}}{2k}-R$$

With higher bargaining power values for developers, the final prices and their corresponding ICERs approach the CET which increases the developers' surplus (profits). Contrarily, low values lower the final prices and corresponding ICERs which reduces the developers' surplus share.

By removing the R&D cost component from Equation 9, we identify the short-term developer surplus curve and reserve ICER. This is captured by:

(11) $$DS^{\prime }=\beta \left[\int_{0}^{\bar{\lambda }}\left(a-\frac{a}{k}\lambda \right)d\lambda -\bar{\lambda }\left(a-\frac{a}{k}\;\bar{\lambda }\right)\right]$$

By rearranging, we have:

(12) $$DS^{\prime }=\frac{\beta a{\bar{\lambda }}^{2}}{2k}$$

Both DS and $DS^{\prime }$ are upward-sloped convex functions, increasing in $\bar{\lambda }$ (the threshold). The shape of DS and $DS^{\prime }$ can be explained in two steps:

1. If there is no entry of new technologies, an increased threshold produces increases of the same proportion in the profit of developers already supplying technologies. This effect guarantees that DS and $DS^{\prime }$ are, at least, linearly increasing in $\bar{\lambda }$.
2. Additionally, assuming we have uniformly distributed the reserve ICERs along the threshold level range [0, *k*], new technologies with reserve ICERs below an increased threshold will enter the market. Profits obtained by these new entrants additionally increase the total industry surplus.

We compare DS and $DS^{\prime }$ to explain how incorporating R&D costs determines the minimum CET that produces a non-negative return for developers.

Equation 12 shows that when R&D cost is not considered, a CET close to zero may be sufficient in providing a non-negative surplus to developers, depending on manufacturing costs. If the R&D investment of the industry (a sunk cost) is incorporated into the developer surplus function (DS curve), the threshold level necessary to produce positive profits for developers is strictly positive. This is calculated by equating Equation 10 to zero and rearranging:

(13) $$\hat{\lambda }=\sqrt{\frac{2kR}{\beta a}}$$

The implication of Equation 13 is that the minimum CET providing a non-negative surplus to developers depends on both the R&D investment required to develop a new medicine and on bargaining power. Higher levels of R&D investment increase the CET needed to avoid the developers making a loss. Higher (lower) developer bargaining power decreases (increases) the CET needed to avoid the developers making a loss. A graphical illustration of how R&D cost and bargaining power affect the developer surplus is provided in Appendix A.2.

We call $\hat{\lambda }$ the dynamic reserve ICER. Below this level, the industry will stop investing in R&D in the long term. Dynamic efficiency is only achieved when the threshold level is set such that *DS* is non-negative or when the CET is higher or equal to $\hat{\lambda }$.

## 3. Results

We begin by presenting and discussing results from the baseline model. We then assess (i) the implications of incorporating R&D costs; (ii) optimising social welfare based on a social welfare function; and (iii) two alternative policy welfare proposals: (a) maximisation of the NPB, subject to achieving dynamic efficiency, i.e. developers getting non-negative surplus, and (b) an “equal distribution” threshold, i.e. payers and developers obtaining an equal share of the surplus. Finally, we illustrate the impact of two potential sources of payer bargaining power: regulation to drive down prices and drug expenditure and the use of tendering to benefit from market competition.

### 3.1. Results from the baseline model

Our baseline model has developers with all bargaining power (*β* = 1), and R&D cost is equal to zero (*R_i_* = 0). We also assume that all developers’ reserve ICERs are uniformly distributed within the range of all their possible values ($\underline{\lambda }\in [0,\;\lambda^{M}]$, where $\lambda^{M}>k$). A uniform distribution of developers’ reserve ICERs is also assumed by Paulden (10) although he recognises the possibility of skewed distributions, with concentrated densities from non-uniform distributions. We discuss the implications of non-uniform reserve ICER distributions in Appendix A.3. The net population benefit of every CET value is represented by an NPB curve.

**Figure 3:**
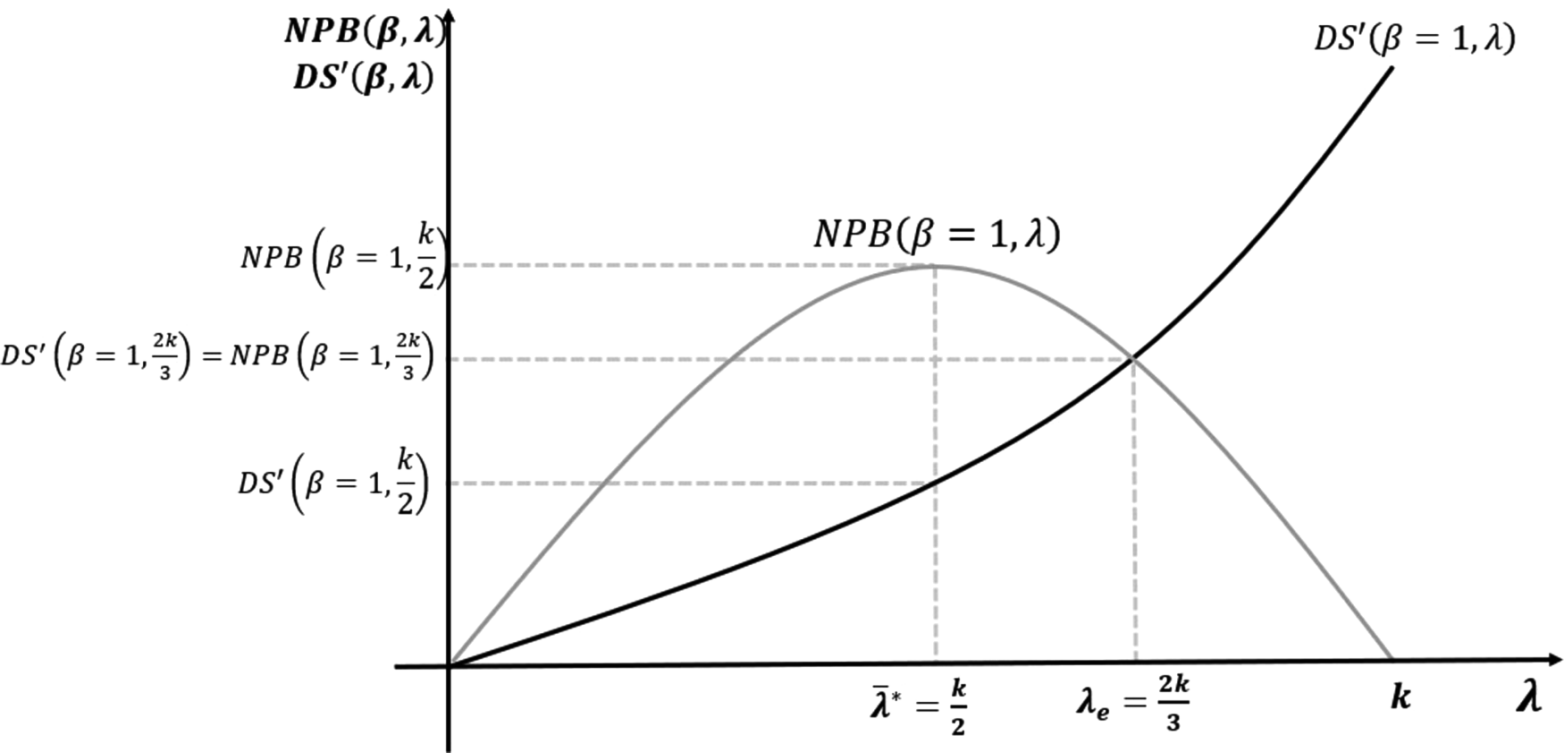
Maximisation of net population benefit (NPB) without research and development (R&D) cost or bargaining power.

We solve the model using backward induction. In Stage 4, the developers decide on ICERs for new medicines, proposing an ICER equal to the CET announced by the payer $\left(\mathrm{ICE}R_{d}=\bar{\lambda }\right)$. In Stage 3, the payer, who is aware of the pricing strategy of the developer, sets the CET to maximise its own surplus $\left({\bar{\lambda }}^{*}=\frac{k}{2}\right)$ as per Equation 8. Stages 2 and 5 are not applicable in the base case because R&D cost and bargaining are not considered. Figure 3 shows the baseline case results with CET set at the maximum of the NPB curve.

In the baseline case, with no R&D cost, the NPB maximising CET is equal to half of the opportunity cost level. Developers react, pricing new medicines where the ICER equals this CET. With no R&D cost, at this NPB maximisation threshold, developer surplus is positive. Medicines with reserve ICERs above the CET are not approved by the payer.

### 3.2. The R&D cost of innovation, reserve ICERs, and dynamic efficiency

Incorporating R&D cost changes the developers’ short-term profit function and long-term reserve ICERs. The static reserve ICER $\underline{\lambda }$ corresponds to price equals marginal cost. The long-term dynamic reserve ICER $\;\hat{\lambda }$ price covers all R&D costs and is the zero-profit price. If the CET level is fixed within the range $\bar{\lambda }\in [\underline{\lambda },\;\hat{\lambda })$, the developers will still supply a developed medicine at a loss rather than losing all R&D costs by not selling. This situation is not sustainable. The developers who expect low or negative returns on R&D investment will stop developing new medicines. The consequence is suboptimal future health innovation if reduced R&D investment for future innovation could be avoided by increasing the CET above ${\bar{\lambda }}^{*}$ while the payer still has a positive or non-negative surplus. To have dynamic efficiency, the CET must be set equal to (or higher than) $\hat{\lambda }$.

**Figure 4:**
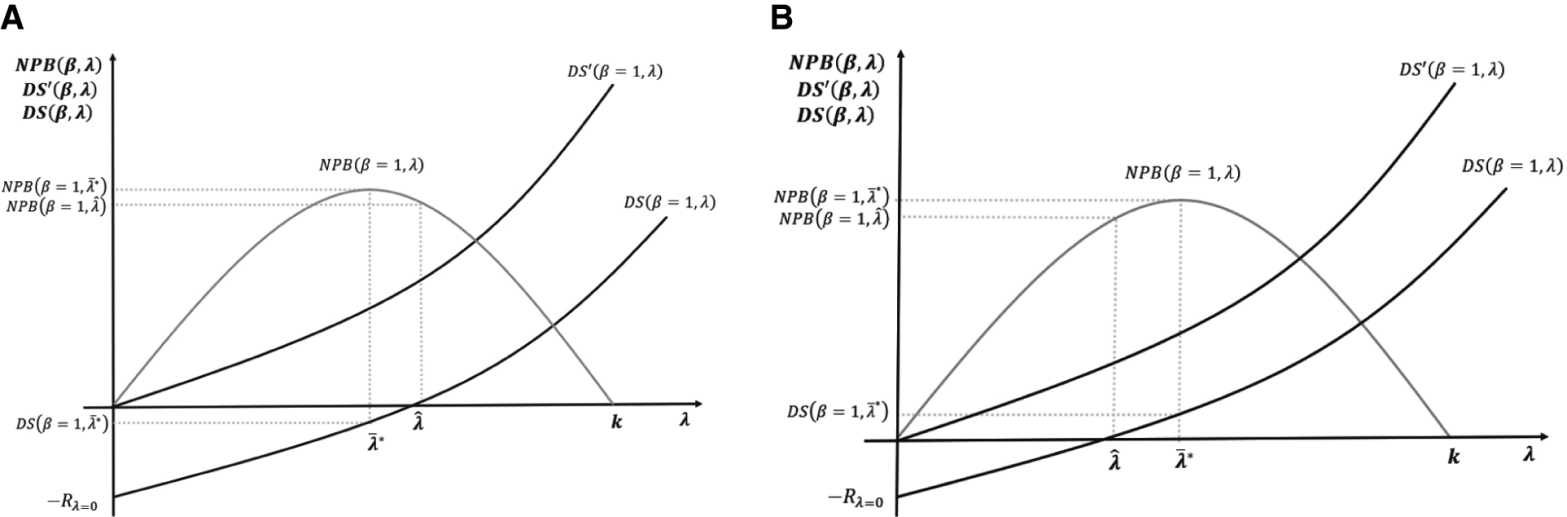
(**A,B**) Maximisation of net population benefit (NPB) with the incorporation of research and development (R&D) cost.

Figure 4A,B illustrates the long-term incentives for the development and supply of new medicines with R&D cost incorporated with two different cases of NPB maximising thresholds.

In Figure 4A, the NPB maximising objective of the payer is not compatible with positive returns for developers. In the short term, medicines are still supplied as price exceeds marginal costs. In the long term, however, low incentives reduce R&D investment. In Figure 4B, the NPB maximising objective provides positive returns for developers; hence, it is incentive-compatible with the long-term R&D investment.

In summary, having introduced R&D cost, we considered the relevant CET for a payer seeking to maximise NPB subject to the constraint of developers having a non-negative surplus. It means setting CET equal to the dynamic reserve ICER of the developers $\hat{\lambda }$.

### 3.3. Maximising social welfare

We now assume that society's objective is to maximise social surplus, defined as the sum of payer and developer surplus as represented in Equations 6 and 10, respectively:

$$\mathrm{SW}(\bar{\lambda })=\mathrm{NPB}(\bar{\lambda })+\mathrm{DS}(\bar{\lambda })$$

Using Equations 6 and 10, the objective function can be rewritten as:

(14) $$argMax_{\bar{\lambda }}(1-\beta )\bar{\lambda }\left(a-\frac{a}{2k}\bar{\lambda }\right)+\beta \bar{\lambda }\left(a-\;\frac{a\bar{\lambda }}{k}\right)+\frac{\beta a{\bar{\lambda }}^{2}}{2k}-R$$

By applying the first-order conditions (the first derivative of Equation 14 equals zero) and rearranging, we get:

(15) $${\bar{\lambda }}^{\mathrm{SW}}=k$$

The optimum threshold from a societal perspective, defined as ${\bar{\lambda }}^{\mathrm{SW}}$, is the payer's maximum ability to pay per unit of health gain (the system opportunity cost *k*).

The socially optimal solution in Equation 15 is the maximum threshold level for which the payer surplus is non-negative. The same result is obtained by Danzon et al. (8, 9) which led them to propose value-based differential pricing as the socially optimal pricing mechanism for the patent period of new medicines assuming the patent period is optimally fixed to allow the payer to capture its share of the surplus after patent expiration. The level of innovation is maximised at the CET corresponding to the payers' (consumers') maximum ability to pay or where the threshold level equals the NPB to zero for every distribution of the bargaining power β∈[0,1].

### 3.4. Alternative social surplus objectives

**Figure 5:**
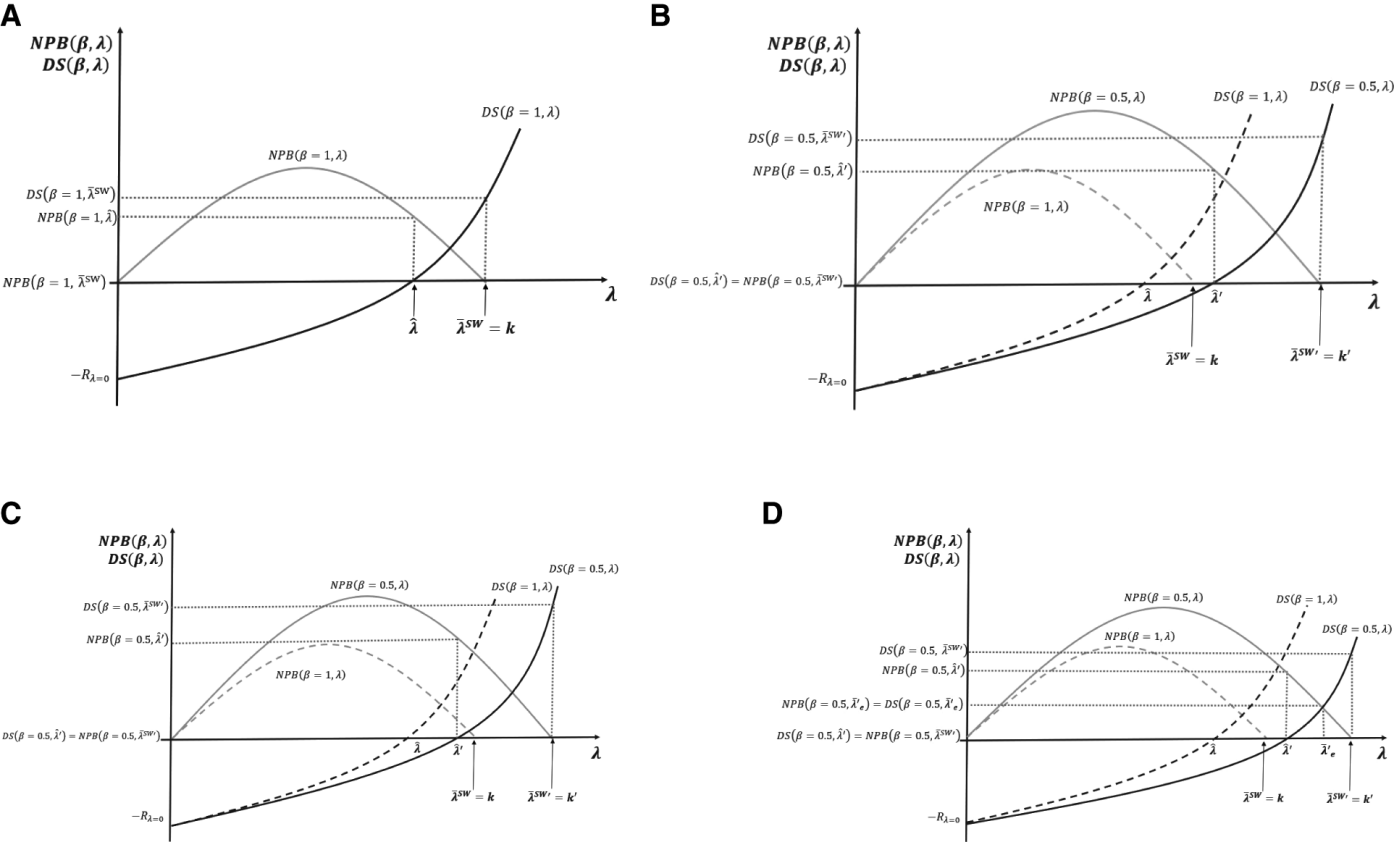
(**A–D**) Cost-effectiveness threshold (CET), social surplus, and dynamic efficiency with contrasting levels of bargaining power.

In Figure 5A–D, we now contrast the potential effects of pursuing three different social surplus objectives: the maximisation of NPB (payer surplus); maximisation of social welfare defined as the sum on NPB and DS, as set out in Section 3.3 above and the equal distribution of surplus between the payer and developers.

Figure 5A presents the case where social surplus is maximised at ${\bar{\lambda }}^{\mathrm{SW}}=k$ with bargaining power all on the developer side *β* = 1, i.e. no bargaining by the payer. The developers accrue all surplus $\mathrm{DS}\;(\beta =1,{\bar{\lambda }}^{\mathrm{SW}})>0$, and the payer obtains $\mathrm{NPB}(\beta =1,{\bar{\lambda }}^{\mathrm{SW}})=0$. Note that if, instead, the payer fixes CET to maximise NPB subject to the developer surplus being non-negative, then the CET is fixed at the dynamic reserve ICER $\hat{\lambda }$. The developer gets $\mathrm{DS}(\beta =1,\hat{\lambda })=0$, and the payer gets the total surplus generated from the resulting level of innovation $\mathrm{NPB}(\beta =1,\hat{\lambda })>0$.

Figure 5B represents the socially optimal result ${\bar{\lambda }}^{\mathrm{SW}\prime }$ when bargaining power is evenly distributed between developers and payers, *β* = 0.5. We reproduce the surplus functions of payers and developers when the latter had all the bargaining power *β* = 1, with new surplus functions, viz. NPB(β=0.5,λ) and DS(β=0.5,λ). In the two cases presented in Figure 5A, ${\bar{\lambda }}^{\mathrm{SW}}$ and $\hat{\lambda }$, we find that in Figure 5B, when bargaining power is *β* = 0.5, the developers' surplus is negative, dynamic efficiency is not achieved, R&D decreases, and long-term innovation is suboptimal. In Figure 5B, efficient CETs are represented by ${\bar{\lambda }}^{\mathrm{SW}\prime }$ for social welfare maximisation and ${\hat{\lambda }}^{\prime }$ for NPB maximisation with the developers' surplus being non-negative. Figure 5B illustrates the values for these two CETs above the original maximum ability to pay off the payer *k*. For the NPB maximising solution subject to the developers' surplus being non-negative, a possible case where ${\hat{\lambda }}^{\prime }<k$ is represented in Figure 5C. In this case, changing the CET to restore dynamic efficiency is not needed.

Figure 5D compares the three different normative solutions when the bargaining power is distributed at *β* = 0.5. We use the situation of Figure 5B as the benchmark. The dynamic efficient ICER with the new distribution of the bargaining power is above the original payer's maximum ability to pay ${\hat{\lambda }}^{\prime }>k$*.* At the social welfare maximising solution, CET $\lambda ={\bar{\lambda }}^{\mathrm{SW}\prime }$, the payer surplus is $\mathrm{NPB}(\beta =0.5,{\bar{\lambda }}^{\mathrm{SW}\prime })=0$, and the developer surplus $\mathrm{DS}(\beta =0.5,{\bar{\lambda }}^{SW^{\prime }})>0$. For the NPB maximising solution subject to the developers' surplus being non-negative, the threshold is $\lambda ={\hat{\lambda }}^{\prime }$. The payer surplus is $\mathrm{NPB}(\beta =0.5,\;{\hat{\lambda }}^{\prime })>0$, and the developer surplus $\mathrm{DS}(\beta =0.5,\;{\hat{\lambda }}^{\prime })=0$. In the equal distribution solution, the condition to set the CET is that both surpluses, the payer and the developer, are equal to $\mathrm{NPB}(0.5,{\bar{\lambda }}_{e}^{\prime })=DS(0.5,{\bar{\lambda }}_{e}^{\prime })$. For that to happen, the CET must be fixed at $\lambda ={\bar{\lambda }}_{e}^{\prime }$. The surplus is non-negative for both parties.

Previous results for the CET level obtained in Equations 8 and 15 and the alternative social surplus objectives discussed in this section compare with the results of Paulden (10) as follows. Equation 8 of this paper shows the maximisation of the NPB from the consumer perspective which corresponds to the “maximise consumer surplus” solution of Paulden (10). The social welfare maximum ${\bar{\lambda }}^{\mathrm{SW}}$ of Equation 15 corresponds to the “maximise producer surplus, subject to producer and consumer surplus being non-negative” solution of Paulden (10). The consumer perspective can also be taken subject to the constraint that producer surplus (DS) is non-negative, which is called “maximise consumer surplus, subject to producer and consumer surplus being non-negative” which coincides with the CET set at dynamic ICER that we have discussed in the previous section when we analysed R&D cost implications. This is also the perspective taken by Woods et al. (12) in their proposal for achieving dynamic efficiency. Finally, our proposal for setting the CET at “equal distribution of surplus” coincides with the particular case of Paulden’s (10) proposal of “maximise consumer surplus, subject to producer surplus comprising a guaranteed proportion of the combined surplus and also subject to each being non-negative”, where the consumer surplus is equal to the producer surplus. However, it is important to note that the implications of the bargaining process and the sunk nature of the R&D costs into the CET level are not captured by Paulden (10).

### 3.5. Market conditions and bargaining power

#### 3.5.1. Imposition of price regulation in addition to thresholds and cost-effectiveness analysis

Payers as policymakers may impose pricing and reimbursement regulation as part of medicine procurement, in addition to using ICER pricing. They aim by pushing down prices to either facilitate access to treatments that are not cost-effective at current prices or to limit expenditure on cost-effective treatments. This exerts payer bargaining power at Stage 5 of Figure 1. These regulations change the effective ICER of the new medicine either by affecting the final price per unit through discounts/caps or total expenditure (revenue) through rebates, clawbacks, or budget limits. This regulation moves the effective ICER of these medicines *λ^E^* to a lower level than the CET used for decision-making $\bar{\lambda }$. Consequently, regulated medicines that accessed the market priced at CET $\bar{\lambda }$ at launch now have a lower price corresponding to an effective ICER of *λ^E^*. This involves a transfer of surplus from the developers to the payer.

**Figure 6:**
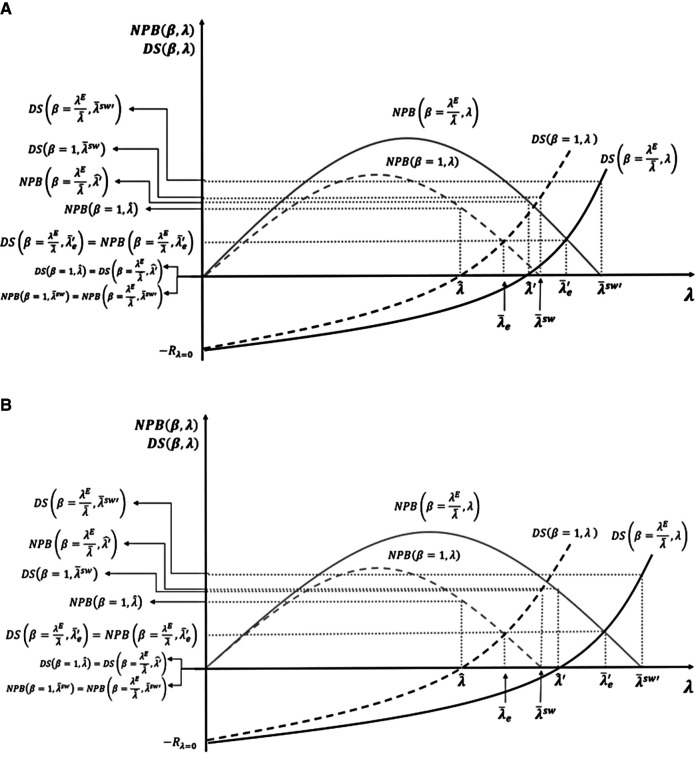
(**A,B**) Regulation, bargaining power, and cost-effectiveness thresholds (CETs).

Figure 6A,B represents the change in the distribution of the surplus and in the dynamic reserve ICER $\hat{\lambda }$ implied by an increase in payer bargaining power. In the previous section, we also illustrated how the three different solutions proposed change in response. We assume that the bargaining power impact of extra regulation is inversely proportional to the change of the effective ICER, $\beta =\frac{\lambda^{E}}{\bar{\lambda }}$. Therefore, the new developer surplus is represented by the curve $\mathrm{DS}\left(\;\beta =\frac{\lambda^{E}}{\bar{\lambda }},\lambda \right)$, and the new payer surplus is represented by the curve $\mathrm{NPB}\left(\beta =\frac{\lambda^{E}}{\bar{\lambda }},\lambda \right)$.

The change in bargaining power is represented in Figure 6A,B by rotation of the DS and NPB curves (from the dashed lines corresponding to *β* = 1 to the continuous lines corresponding to $\beta =\frac{\lambda^{E}}{\bar{\lambda }}$. In this figure, we present the three solutions: NPB maximisation subject to the developers' surplus being non-negative $\left(\hat{\lambda }\right)$; equal distribution $\left({\bar{\lambda }}_{e}\right)$; and social welfare maximisation $\left({\bar{\lambda }}^{\mathrm{SW}}\right)$. In Figure 6A, the social welfare maximising solution remains dynamically efficient after the change in the bargaining power $\left(k={\bar{\lambda }}^{\mathrm{SW}}>{\hat{\lambda }}^{\prime }\right)$ while the other two solutions are now dynamically inefficient $\left(\hat{\lambda }<{\bar{\lambda }}_{e}<{\hat{\lambda }}^{\prime }\right)$. The CET levels to implement the same three solutions after the regulation are represented in the figure by ${\hat{\lambda }}^{\prime },{\bar{\lambda }}_{e}^{\prime }$, and ${\bar{\lambda }}^{\mathrm{SW}\prime }$, with the new equal distribution and social welfare maximising solutions being higher than the original maximum ability to pay off the payer $\left(k={\bar{\lambda }}^{\mathrm{SW}}\right)$.

In Figure 6B, none of the three solutions corresponding to *β* = 1 are dynamically efficient under bargaining power distribution $\beta =\frac{\lambda^{E}}{\bar{\lambda }}(\hat{\lambda }<{\bar{\lambda }}_{e}<{\bar{\lambda }}^{\mathrm{SW}}<{\hat{\lambda }}^{\prime })$. At a minimum, to restore dynamic efficiency and long-term R&D investment in innovation, the CET should be fixed at NPB maximisation subject to the developer surplus being non-negative. That CET, unlike in Figure 6A, is located above the original payer's maximum ability to pay $\left(k={\bar{\lambda }}^{\mathrm{SW}}<{\hat{\lambda }}^{\prime }\right)$.

#### 3.5.2. Using competition in markets for patented pharmaceuticals

Market competition affects the distribution of bargaining power. Our focus is “in-patent” price competition for patented medicines from new alternative medical therapies occurring in many diseases or conditions (Berndt et al. (13); Kanavos et al. (14); Danzon and Chao, (15)). Examples include direct-acting antiviral treatments for hepatitis C (Roediger et al. (16); Berdud et al. (17)), second-generation antipsychotics (18), and oncologic medicines (Vokinger et al., (19)).

If the payer implements the mechanisms such as tendering, it can use competition to increase the bargaining power and impact prices at the procurement stage (Stage 5 of Figure 1). The effective ICERs will be lower than those used in the initial decision-making, changing the effective surplus distribution between the payer and developers.

**Figure 7:**
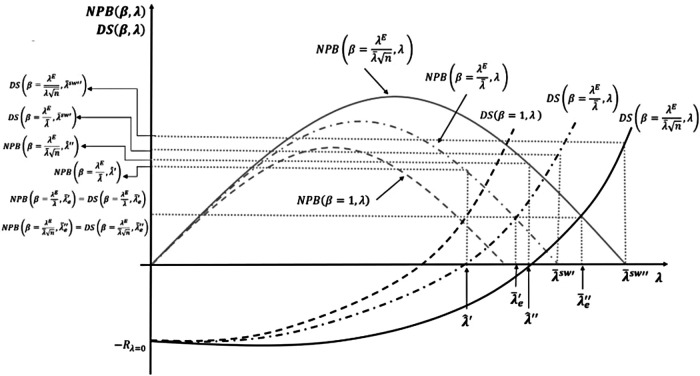
Competition, bargaining power, and cost-effectiveness thresholds (CETs).

To make the bargaining power in our model depend on market competition, we add it to the enhancement of bargaining power arising from regulation, $\frac{\lambda^{E}}{\bar{\lambda }}$, with the following function:

(16) $$\beta =\frac{\lambda^{E}}{\bar{\lambda }\sqrt{n}}$$

where *n* represents the number of substitute medicines and the bargaining power of the payer (1−β) positively depends on competitor numbers *via* $\;\sqrt{n}$. Figure 7 shows how competition changes the surplus distribution by increasing payer bargaining power in addition to the increase from regulation.

Figure 7 compares our three results: NPB maximisation subject to the developers' surplus being non-negative, the equal distribution solution, and the social welfare maximising solution. It shows the effects when bargaining power is introduced first by regulation and then, additionally, by competition. With increased payer bargaining power due to competition, the CET for the NPB maximisation is subject to the developers' surplus being non-negative increases from ${\hat{\lambda }}^{\prime }$ to ${\hat{\lambda }}^{\prime \prime }$. Not adjusting the CET to ${\hat{\lambda }}^{\prime \prime }$ when competition plays a role means the developers' surplus can turn negative. Figure 7 also shows that the equal distribution solution CET increases from ${\lambda \prime }_{e}$ to $\lambda_{e}^{\prime \prime }$, when a competition effect on bargaining is introduced. If the CET is not adjusted to $\lambda_{e}^{\prime \prime }$, the developers' surplus can turn negative. Finally, we compare the social surplus maximising solution with and without competition. Competition increases the social welfare maximising CET from ${\bar{\lambda }}^{\mathrm{SW}\prime }$ to ${\bar{\lambda }}^{\mathrm{SW}\prime \prime }$. In this case, the CET level ${\bar{\lambda }}^{\mathrm{SW}\prime }$ still guarantees a positive level of surplus to developers and so is dynamically efficient, but the social surplus is not maximised.

## 4. Discussion

We develop a general theoretical model of ICER pricing and CET decision-making, using game theory to assess the distribution of the social surplus generated by new health technologies. Our baseline model uses the assumptions of Paulden (10). Paulden (10) models the CET accounting for (i) developers’ strategic behaviour and pricing of new technologies where the ICER equals the threshold and (ii) the impact of CET on consumer and producer surplus. Using a set of assumptions, the model produces policy proposals for determining the CET which differ depending on who's surplus is maximised—payer or developer's—and which constraints are applied. For example, policy proposals to set the CET level to maximise payer or producer surplus can be complemented with constraints such as the other party's surplus being non-negative or the other party being given a share of the total surplus (10).

We incorporate three additional elements to Paulden (10). The main addition is the incorporation of bargaining analysis using NBS, crucial when analysing single buyer (monopsonist) third-party payer systems and innovation developed by patent-protected sellers (monopolists). A second crucial addition of our model to Paulden (10) is the formal incorporation of (sunk) R&D cost enabling discussion of the long-term supply of innovation versus incentives for developers who have a new medicine approved to sell in the short-term at prices that do not cover full R&D costs. Thirdly, we incorporate non-uniform distributions of the developers' reserve ICERs leading to non-linear developer surplus curves (see Appendix A.3). This case, although recognised by Paulden (10) (see *Further considerations, p. 14*) was not analysed. We develop a non-uniform distribution of developers’ reserve ICERs to assess the implications for surplus distribution and CET setting. We consider this needs future research because it is crucial to understanding the endogenous relationship between the CET level and the degree of scientific challenge and risk accepted at the margin by developers. For example, by increasing the ICER, supply will increase because developers will fund riskier and more challenging projects with higher expected returns. This produces a non-linear relationship between the CET and supply (reserve ICERs).

We show that if R&D costs are considered the developers' surplus can turn negative (loss-making) for a range of positive threshold values. In the short term, developers will supply already-developed new medicines, subject to prices exceeding marginal costs, to get some contribution to sunk R&D costs. This is statically efficient, as it maximises access to (already developed) medicines. However, it is not dynamically efficient, as it disincentivises future innovation. We show that the CET that maximises the NPB should be set such that developers achieve a non-negative long-run surplus, which we term the dynamic reserve ICER. Any CET level below this dynamic reserve ICER generates low investment in future innovation with an overall health loss for society. However, setting such a CET level requires accurate knowledge of supply CET elasticity and by implication how developers’ reserve ICERs are distributed for different CET values. Our analysis of the non-uniform distributions of the reserve ICERS (see Appendix A.3) is crucial to explore how the degree of scientific challenge (or risk) of the marginal increases with an increased CET. We identify three implications: (i) the relationship between the technical difficulty and the risk of pharmaceutical innovation undertaken and the threshold level is endogenous; (ii) pharmaceutical innovation supply is increasing at the marginal CET level; and (iii) it is likely that reserve ICERs for some desired breakthrough innovations are above the CET and policymakers need to find ways to incentivise it, by using CET or another mechanism.

Incorporating bargaining using NBS enables the analysis of a payer’s use of non–value-based regulation and the impact of competition to negotiate prices for approved new medicines lower than the CET used for decision-making. This changes surplus distribution, increasing the maximum ability to pay of the system. When payers have most or all bargaining power, we find that, perhaps somewhat counterintuitively, the maximum acceptable CET level for initial decision-making will be higher than the system's opportunity cost level. This is mutually beneficial for payers and developers, as the payer will access more innovation and be able to obtain a positive surplus (net health gains) from them due to its increased bargaining power to negotiate prices after approval. This needs consideration by policymakers. These effects are complex and technically difficult to incorporate at the stage when the CET level for decision-making must be set. They need, however, to be considered somehow by HTAs at the assessment stage if optimal outcomes are to be achieved. A focus of policymakers should be to understand how the final effective ICERs of medicines are determined in practice and to use that information to find decision-making CETs that maximise social surplus. To that end, further analysis and empirical research are needed.

We model the proportions of surplus produced in the long term given to each party. Paulden (10) argued that the focus must be the maximisation of the NPB, ignoring developer surplus. We show that this objective is inefficient unless it is conditioned on producer surplus being non-negative. We also show that if other objectives such as social surplus maximisation or achieving an equal distribution of the surplus are pursued, alternative levels of CETs for decision-making have to be explored.

The study of the optimal threshold level from a social welfare perspective also requires a multistage modelling approach. In the single patent-protected time period approach presented in this paper, we establish that the socially optimal threshold is the maximum ability to pay of the payer—or the health system opportunity cost. This threshold level gives all the surplus to the industry. In their work, Danzon et al. (8, 9) showed that this is the socially optimal result assuming an optimal patent length, after which payers capture most of the surplus through generic or biosimilar competition.

Overall, the results depend on the marginal productivity of the health system (the maximum ability to pay for innovation) and the payoff functions of the payer (NPB) and developers (DS). These functions can be significantly affected by bargaining power, such as competition and the use of non–value-based procurement regulation, and by the size and distributions of R&D costs (reflected in reserve ICERs). We show that under some circumstances, these factors can increase the HTA decision-making CET of the payer above the health system's opportunity cost while remaining incentive-compatible for all stakeholders in the market.

We identify several areas for future research, including understanding the long-term supply elasticity of R&D response to changes in the CET. A critical omission in this paper is two-part modelling over two periods: pre- and post-patent expiry. We follow Paulden (10) by assuming an infinite patent life or by focusing exclusively on the patent period. Post-patent expiry price evolution has a significant impact on both the size of the surplus generated and on its distribution (12–14) with generic and biosimilar competition.

## 5. Conclusion

Health systems around the world are increasingly using HTA assessments to inform decisions about reimbursement. These often include ICER-based cost-effectiveness analysis with decision-makers using the CET as a pricing tool.

We developed a model of ICER pricing allowing us to understand the implications for the optimal level of CET of the amount of and distribution of social surplus generated by new medicines. The model incorporated relevant aspects such as the bargaining power, (sunk) R&D cost, and non-uniform distribution of developers’ reserve ICERs.

Incorporating R&D cost introduces a dynamic aspect. The CET that maximises short-term population health benefits may not be optimal in the long term. Policy decision-makers should always set the CET equal to or above the dynamic reserve ICER of developers, which requires greater understanding as to where the dynamic reserve ICER is located and the elasticity of responsiveness of R&D to changes in the CET, i.e. the distributions of R&D costs as reflected in reserve ICERs.

Bargaining is incorporated to reflect how market functioning and regulation can change surplus distribution and optimal CET. With a large payer bargaining power, the efficient HTA decision-making CET to maximise short- and long-term health gains may be above the system opportunity cost. Decisions around an optimal HTA decision-making CET need to be informed by better knowledge about how real-world bargaining power distribution affects the effective levels of new health technologies' ICERs.

We conclude that it is not evident that using a certain fixed, static level of CET in HTA decision-making as a binding condition for new medicine reimbursement is optimal for society. Flexible interpretation of the CET applied to account for the factors discussed in this work should be considered, supported by further research on how policy and market functioning affect the distribution of bargaining power and resulting surpluses and more accurate knowledge about how R&D innovation and investment respond to rewards, i.e. the elasticity of innovation.

## Figures and Tables

**Figure A1:**
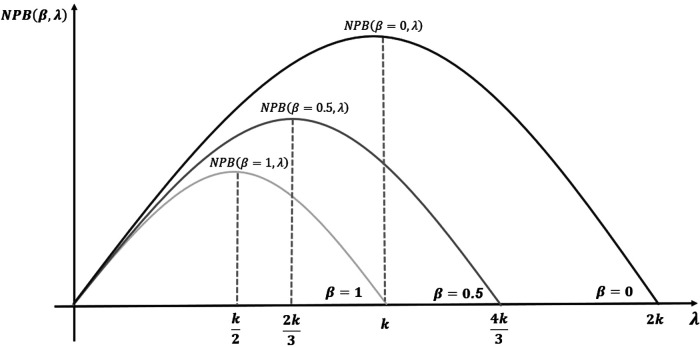
The payer surplus (NPB) by bargaining power and CET.

**Figure A2:**
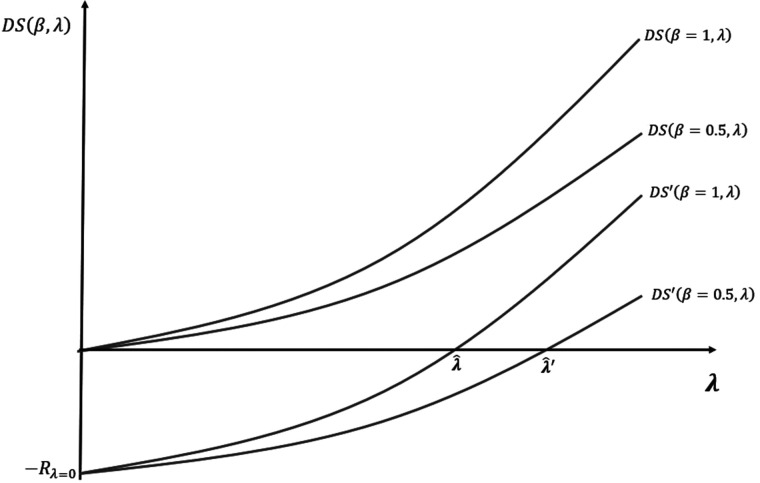
Shape of the DS and DS′ curves for different bargaining power. $-R_{\lambda =0}$ is the loss of R&D of the industry at a threshold level equal to zero.

**Figure 8:**
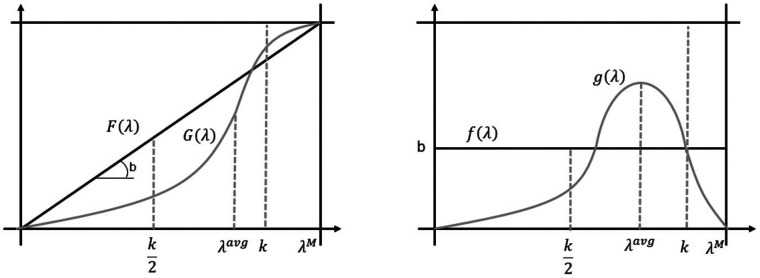
Non-uniform distribution of reserve incremental cost-effectiveness ratios (ICERs).

**Figure 9:**
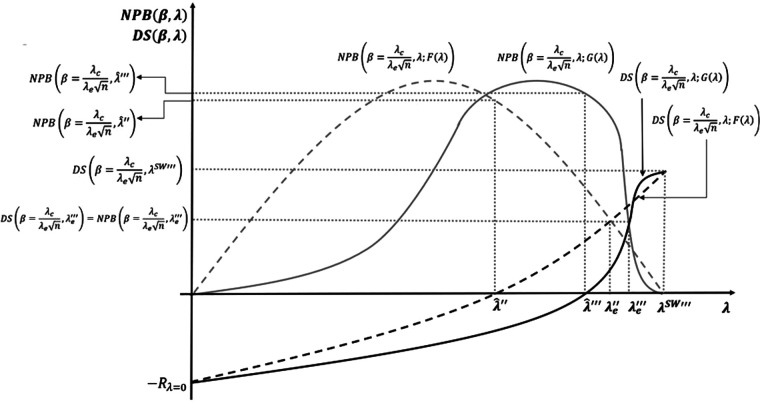
Non-uniform distribution of reserve incremental cost-effectiveness ratios (ICERs) and cost-effectiveness thresholds (CET).

## Data Availability

The original contributions presented in the study are included in the article/Supplementary Material; further inquiries can be directed to the corresponding author.

## Appendix

### A.1. The micro-bargaining model

This appendix develops formally the payoff function of the developer, the payoff function of the payer, the Nash bargaining solution for the drug price bargaining game, the bargaining set, and the total surplus as the sum of the consumer (payer) and the producer (developer) surpluses which depend on the bargaining power distribution.

#### A.1.1. The developer

Let the profit function of the developer of medicine *i* be:

(A1) $$\Pi (\;p_{i})=p_{i}q_{i}-C_{i}(q_{i})$$

where *q_i_* is the number of patients treated (the intention-to-treat population), which is taken as given; *p_i_* is the final price per unit of medicine; and $C_{i}(q_{i})$ is the production cost function of the developer which we define as:

(A2) $$C_{i}(q_{i})=R_{i}+cq_{i}$$

where *R_i_* represents the R&D cost incurred by the developer to develop medicine *i*^1^ and *cq_i_* is the variable cost the developer needs to incur to sell *q_i_* units of medicine *i*, with *c* being the marginal cost of production. The R&D cost is a sunk cost; hence, the developer has incentives to sell the medicine in the short run starting at a price that provides a benefit higher than −*R_i_*. This level of price is determined by solving Equation A3:

(A3) $$-R_{i}\leq p_{i}q_{i}-(R_{i}+cq_{i})$$

By rearranging, we have:

$$\underline{\;p}\geq c$$

However, in the long run, the developer does not have incentives to develop the medicine if the final price does not (at a minimum) ensure the R&D cost—in addition to the cost of manufacturing^2^—will be recovered. This long-run price is calculated by solving Equation A4:

(A4) $$0\leq p_{i}q_{i}-(R_{i}+cq_{i})$$

By rearranging, we have:

$$\hat{\;p}\geq c+\frac{R_{i}}{q_{i}}$$

We now define the developer’s short-run and long-run reserve ICERs. First, let the developer's ICER of a new medicine is defined as:

(A5) $$\mathrm{ICE}R_{d}=\frac{\;p_{i}-p_{j}}{h_{i}-h_{j}}$$

where *p_j_* is the price of the comparator (usually the standard care at the time of the adoption of the new medicine *i*; *h_i_* and *h_j_* are the health gains per patient of the new medicine and the comparator, respectively^3^). As the ICER is defined as a function of the price of the medicine, we can define the short- and long-run reserve ICERs. Let $\underline{\lambda }$ be the short-run reserve ICER of the developer, which corresponds to a price level $p_{i}=\underline{\;p}$, and let $\hat{\lambda }$ be the long-run reserve ICER of the developer, which corresponds to a price level $p_{i}=\hat{\;p}$. In addition, assuming that the patient population is fixed, the profit of the developer can also be expressed as a function of the ICER, where acceptable profits are only achieved with ICERs equal to or above the long-run reserve ICER, that is, $\lambda_{i}\geq \hat{\lambda }$, where $\lambda_{i}$ is the ICER corresponding to the final price *p_i_*.

Finally, we define the outside option of the developer—denoted as $\Theta^{d}$—as the payoff to the developer where an agreement on a price is not successfully reached. We assume that there are no private health markets and no out-of-pocket demand for the medicine. Thus, the outside option of the developer, which is conditioned on having developed the product, is a negative profit equal to the magnitude of the sunk R&D cost:

(A6) $$\Theta^{d}=-R_{i}$$

#### A.1.2. The payer

In Stage 1, nature sets the maximum ability to pay the payer of the threshold λ={k,υ}k which determines the maximum amount payable per unit of health gain. Let us assume for now that the payer fixes the CET level at the level of its maximum ability to pay. The (gross) payoff function of the payer is then:

(A7) $$B_{i}(\;p_{i})=\lambda h_{i}q_{i}-(\;p_{i}q_{i}+T_{i}(q_{i}))$$

where the first addend on the right-hand side of Equation A7 represents the monetary value of total health gains obtained by patients treated with medicine *i*. The second addend—in parenthesis—represents the total cost to the system of treating patients with medicine *i*. This includes both the drug cost (*p_i_q_i_*) and the direct cost for the health system $T_{i}(q_{i})$, e.g. visits to the GP, drug administering cost, inpatient days, and emergency visits.

To define the reserve price *p* (the maximum price that the payer is willing to pay for the medicine *i*) and the corresponding reserve ICER *λ* at that price, we need to first define the outside option of the payer $\Theta^{p}$. This outside option is the payoff to the developer where a price *p_i_* for the medicine *i* is not successfully agreed upon, or the payoff obtained by treating *q_i_* patients with an existing medicine *j* (the comparator). Equation A8 represents the outside option of the developer:

(A8) $$\Theta^{p}=B_{j}(q_{i})=\lambda h_{j}q_{i}-(\;p_{j}q_{i}+T_{j}(q_{i}))$$

By calculating $B_{i}(q_{i})-\Theta^{p}$, we measure the net monetary value (the surplus) of the health gain, or the net population benefit (NPB) obtained by the payer from using medicine *i*, as follows:

(A9) $$\mathrm{NP}B_{i}(\;p_{i})=B_{i}(\;p_{i})-\Theta^{p}=\;\lambda (h_{i}-h_{j})q_{i}-(\;p_{i}-p_{j})q_{i}-(T_{i}(q_{i})-T_{j}(q_{i}))$$

The system will then adopt the new medicine whenever $\mathrm{NP}B_{i}(q_{i})\geq 0$, that is, when the monetary value of the incremental health benefit exceeds the incremental cost. We define the exact cut-off point by equating Equation A9 to zero in Equation A10:

(A10) $$B_{i}(q_{i})-\Theta^{p}=0$$

Again, assuming that $T_{j}(q_{i})=T_{i}(q_{i})$, then substituting Equations A7 and A8 in Equation A10 and rearranging, we obtain:

(A11) $$\lambda =\frac{\;p_{i}-p_{j}}{h_{i}-h_{j}}$$

This corresponds to a price *p_i_* = *p*, which is the reserve price of the payer given the reserve ICER for the payer of *λ*.

#### A.1.3. The bargaining problem between the developer and the payer

At Stage 2, before the game reaches the bargaining process (Stage 5), the payer commits to a maximum threshold level $\bar{\lambda }\in [0,\lambda ]$, which determines their reserve ICER. If the short-run reserve ICER of the developer is lower than the reserve ICER of the payer $\left(\underline{\lambda }<\bar{\lambda }\right)$, then both parties can agree on a final price of the medicine *p_i_* which is related to a particular ICER *λ_i_* level such that $\underline{\lambda }\leq \lambda_{i}\leq \bar{\lambda }$. Such an agreement secures non-negative profits to the payer and a loss of a smaller size than the sunk cost $-R_{i}$ to the developer. Therefore, in a model of value-based pricing with an explicit (or implicit) CET, price negotiations at the health system's different contracting levels are equivalent to negotiations over the ICER *λ_i_* level. The result of this bargaining process is a price for the medicine and its corresponding ICER, i.e. the effective ICER.

We follow the Nash bargaining approach to model the final accepted ICER (or final price) negotiation. The Nash bargaining solution divides the total profit of an economic interaction between negotiating parties in such a way that the product of all players’ net benefit over the disagreement point is maximised for a given value of their bargaining power. In economic negotiation contexts where players’ “impatience” plays a key role in the final outcome, the Nash bargaining solution is an efficient approach for modelling (15). This is the case in pharmaceutical markets where the payers’ impatience is defined by the clinical need and developers’ impatience is defined by the sunk nature of R&D cost. The Nash bargaining solution has been applied to model pharmaceutical markets in different settings (16–19).

In the context of our model, the agreed ICER net level from the reserve ICER determines the share of the pie each player obtains. The Nash bargaining approach over the ICER level can be represented as:^4^

(A12) $$\mathrm{argMa}x_{\lambda_{i}}\;(\lambda_{i}-\underline{\lambda }{)}^{\beta }(\;\bar{\lambda }\;-\lambda_{i}{)}^{1-\beta }$$

where *β* is the bargaining power of the developer, (1−β) is the bargaining power of the payer, and αϵ[0,1]. By solving Equation A12 with the first-order conditions being applied, we obtain *λ_i_* as a function of *β*. As the price is a function of the agreed ICER, *p_i_* is also obtained. Substituting *p_i_* into Equations A1 and A9, we obtain the payoffs of the developer and the payer, respectively.

By solving Equation A12, we obtain the Nash bargaining solution of the ICER:

(A13) $$\lambda_{i}^{*}=\beta \bar{\lambda }+(1-\beta )\underline{\lambda }$$

where $\lambda_{i}^{*}\in [\underline{\lambda },\;\;\bar{\lambda }]$ with $\frac{d\lambda_{i}^{*}(\beta )}{d\beta }>0;\Pi (\;p_{i})\in [0,\Pi (\bar{\;p})]$ with $\frac{d\Pi (\;p_{i}(\lambda_{i}^{*}))}{d\lambda_{i}^{*}(\alpha )}>0$; and $B(\;p_{i})\in [0,B(\underline{\;p})]$ with $\frac{dB(\;p_{i}(\lambda_{i}^{*}))}{d\lambda_{i}^{*}(\alpha )}<0$.

#### A.1.4. Delimiting the set of possible bargaining outcomes and agreements

To analyse possible solutions to the bargaining process and the economic and social welfare implications of ICER pricing at the micro-level, we establish the following set of assumptions which allow us to simplify the payoff functions of both the payer and the developer and show how bargaining power affects both parties’ benefits and social welfare:

1. We normalise incremental cost and incremental health benefit by assuming $h_{j}=0$, $p_{j}=0$ and $T_{j}(q_{i})=0$.
2. The new medicine always produces positive incremental health benefits: $h_{i}>0$.
3. The new medicine is always served at positive incremental cost: $\underline{\;p}>0$.
4. Let the direct healthcare cost of using medicine *i* be equal to the direct healthcare cost of using medicine $j:T_{i}(q_{i})=T_{j}(q_{i})=0$.
5. Let the total treatment patient population be $q_{i}=Q$.

Following the ICER definition in Equation A5 and applying the assumptions above, we can now define the price as a function of the ICER as in the following equation:

(A14) $$p_{i}(\lambda_{i})=\lambda_{i}h_{i}$$

By substituting Equation A14 into Equations A1 and A9 and applying all assumptions, we have the following payoff functions for the developer and the payer, respectively:

(A15) $$\Pi (\lambda_{i})=(\lambda_{i}h_{i}-c)Q-R_{i}$$

(A16) $$\mathrm{NPB}(\lambda_{i})=(\lambda -\lambda_{i})h_{i}Q$$

Figure 2 graphically delimits the bargaining set—that is, all potential agreements over the final ICER $\lambda_{i}^{*}$ that are incentive-compatible (or provide mutual positive payoffs) for both the payer and the developer. The possible mutually beneficial agreements are all *λ_i_* values such that $\lambda_{i}\in [\underline{\lambda },\;\bar{\lambda }]$, where $\;\bar{\lambda }<\lambda$. Figure 2 shows that for *λ_i_* values such that $\lambda_{i}\in [\underline{\lambda },\hat{\lambda }]$, the profit of the developer is negative but higher than sunk costs $\left(-R_{i}<\Pi <0\right)$. For *λ_i_* values such that $\lambda_{i}\in [\hat{\lambda },\;\bar{\lambda }]$, the payoff to the developer is zero or higher $\left(0\leq \Pi \leq \Pi (\bar{\lambda })\right)$.

Introducing the Nash bargaining solution (A13) into both Equations A15 and A16, we obtain the following:

(A17) $$\Pi (\lambda_{i}^{*})=((\beta \lambda +(1-\beta )\underline{\lambda })h_{i}-c)Q-R_{i}$$

(A18) $$\mathrm{NPB}(\lambda_{i}^{*})=(\left(1-\beta )(\bar{\lambda }-\underline{\lambda })+(\lambda -\bar{\lambda }\right))h_{i}Q$$

where the total surplus $\mathrm{TS}(\lambda_{i}^{*})$ at the micro-level—one payer and one developer—is given by the sum of Equations A17 and A18:

(A19) $$\mathrm{TS}(\lambda_{i}^{*})=\Pi (\lambda_{i}^{*})+\mathrm{NB}(\lambda_{i}^{*})$$

### A.2. Illustration of the bargaining power and R&D cost effects on the payer’s and developers’ surpluses

#### A.2.1. Bargaining power effect on the payer surplus

Table A1 shows the $\lambda^{*}$ values and maximum ability to pay off the payer, expressed in terms of the supply-side CET *k* and for different bargaining powers *β*. Table A1 also includes the value of the developer's bargaining power that leads to the scenario of $\lambda^{*}=k$.

**Table A1:** Surplus maximising CET and WTP for the payer by bargaining power.

Developer: *β*	Payer: 1 − *β*	Optimal payer CET: $\lambda^{*}$	Maximum WTP
1	0	*k*/2	*k*
3/4	1/4	4*k*/7	8*k*/7
1/2	1/2	2*k*/3	4*k*/3
1/4	3/4	4*k*/5	8*k*/5
0	1	*k*	2*k*

Figure A1 illustrates how changes (of different magnitudes) in the relative bargaining power of the payer and the developer affects the NPB curve. The payer surplus is maximised at higher ICER values when their own bargaining power increases and the bargaining power of the developer decreases. The maximum WTP also increases when the payer's bargaining power increases. This is because, when the threshold is increased to obtain new technologies, the extra amount paid for non-marginal existing interventions will be lower.

#### A.2.2. Bargaining power and R&D cost effects on the payer surplus

Figure A2 illustrates the relationship between *λ*, R&D costs, and developers’ surplus. To examine how bargaining power affects the *DS* curve and its graphical shape, we look at the value of β∈[0,1]. As it is a non-negative number with lower and upper bounds at zero and one, respectively, it is effectively a proportion. The *DS* increases/decreases proportionally with higher/lower *β* values. With changes in the bargaining power, the *DS* curve pivots over the origin (−*R* when *λ* = 0). It pivots down (up) when *β* decreases (increases).

This is because more bargaining power means a greater ability to extract surplus by pricing new medicines closer to the threshold. However, when payers have some bargaining power, the agreed prices of new medicines after the procurement and commercial stage may fall below the threshold and part of the developers’ profit will be extracted by the payer. In such a situation, to avoid dynamically inefficient market outcomes, the threshold should be increased until the condition in (12) is restored. This is shown in Figure A2 by the values $\hat{\lambda }$ and ${\hat{\lambda }}^{\prime }$ which are the threshold levels that ensure dynamic efficiency for the developer's bargaining power values, *β* = 1 and *β* = 0.5, respectively. Figure A2 shows that with a lower (higher) bargaining power of the developer (payer) *β* = 0.5, the value of the threshold that ensures dynamic efficiency is higher ${\hat{\lambda }}_{d,\beta =0.5}>{\hat{\lambda }}_{d,\beta =1}$.

### A.3. Appendix: the effect of skewed distributions of the reserve ICERs

To introduce skewed distributions of reserve ICERs in the analysis, we rewrite the relationship between the net health gain and the CET as follows:

(A20) $$h(\bar{\lambda })=a-aF(\underline{\lambda })$$

where $\underline{\underline{\lambda }}$ is a variable representing the reserve ICERs and is independent and identically distributed on some interval $\underline{\lambda }\in [0,\lambda^{M}]$ and according to the distribution function $F(\underline{\lambda })$. Apart from the introduction of the distribution function, Equation A20 reflects the same relationship between the CET and the net health gain of the payer calculated in Equation 4 of the main body of the article. For every CET level, it measures the marginal health gain for the system by adopting innovations at ICERs equal or lower to the system's opportunity cost. The form of $h(\bar{\lambda })$ is determined by the underlying technological possibilities, the health production function of the system and by the distribution of the developers’ reserve ICERs. The relationship between net health gain and threshold value can be linear or non-linear and is determined by the underlying distribution of the developers’ reserve ICERs.

The baseline model implicitly assumes a uniform reserve ICER distribution function. It implies that the marginal response of the supply to any change in the CET level is constant. An increased CET level of a constant size incentivises an equal number of additional new medicines to enter the market irrespective of the baseline CET level In this appendix, we explore non-uniform distributions of the reserve ICERs in two cases. Firstly, we assume the best existing technology for developing a type of innovative medicine may be in great part common knowledge and accessible to all developers. It may also be the case where the scientific challenge and (correlatively) the R&D cost are common or similar for certain therapy areas instead of being firm-specific. The reserve ICERS in these cases concentrate on certain ICER values. Secondly, reserve ICERs can concentrate at more than one CET value. Some types of technologies or disease areas may entail projects of higher scientific challenge or risk, implying that the benefit of increasing the CET to incorporate those with higher reserve ICERs, e.g. riskier or more challenging projects, can overcome the cost of paying more for those concentrated at a lower CET level.

To represent this supply concentration around specific ICER values, we assume the reserve ICERs follow a distribution function with mean $\mu =\lambda^{\mathrm{avg}}$, located above the CET level that maximises NPB $\left(\lambda^{\mathrm{avg}}>\lambda^{*}\right)$, and are associated with a skew normal distribution and density functions^5^. An example of a non-uniform distribution is shown in Figure 8. The graph on the left-hand side of the figure compares the uniform distribution F(λ) with a hypothetical skew normal distribution G(λ). The graph on the right-hand side compares the density functions associated with each of the two distributions.

With a distribution G(λ), the market entry point of most of the developers concentrates around a narrow range of ICER values with average $\lambda^{\mathrm{avg}}$. For CET values out of the range, the response of the supply to a change in the CET is small. Alternatively, for CET values within the range, the supply is very sensitive to the change. A small decrease (increase) of the CET within the concentration range of the supply produces a large decrease (increase) in innovative health technologies entering the market and health gains accrued by the system. Therefore, it is important from the policymaking perspective to understand the shape and responsiveness of the supply. For a NPB maximising payer, knowing where exactly the NPB curve reaches its peak and how much responsive it is to changes in the CET is crucial. Such a piece of knowledge requires a better understanding of the underlying distribution of the developers’ reserve ICERS. Figure 9 shows this relationship between the distribution of the reserve ICERs and the decision of setting the CET.

The example in the figure represents a distribution of the reserve ICERS skewed to the right. The benchmark in the example is the model with both, the R&D cost, and the bargaining effects included. A first implication is that the CET level that maximises the NPB subject to the developers’ surplus being non-negative increases from ${\hat{\lambda }}^{\prime \prime }$ to ${\hat{\lambda }}^{\prime \prime \prime }$ when the distribution of reserve ICERs is non-uniform. The SET for the equal distribution solution also increases from $\lambda_{e}^{\prime \prime }$ to $\lambda_{e}^{\prime \prime \prime }$. However, the social welfare maximising solution for the non-uniform distribution $\lambda^{{\mathrm{SW}}^{\prime \prime \prime }}$ remains the same as the uniform distribution, as the distribution of reserve ICERs (the price elasticity of the supply) does not change the maximum that the system is willing to pay for a health gain.
